# The Winding Roadmap of Biomarkers Toward Clinic: Lessons from Predictors of Resistance to Anti-EGFRs in Metastatic Colorectal Cancer

**DOI:** 10.3390/ijms19082298

**Published:** 2018-08-05

**Authors:** Carlotta Antoniotti, Elena Ongaro, Alfredo Falcone, Chiara Cremolini

**Affiliations:** 1 Department of Oncology, University Hospital of Pisa, 56126 Pisa, Italy; carlottantoniotti@gmail.com (C.A.); eleongaro@gmail.com (E.O.); alfredo.falcone@med.unipi.it (A.F.); 2 Department of Translational Research and New Technologies in Medicine, University of Pisa, 56126 Pisa, Italy; 3 Department of Oncology, Azienda Sanitaria Universitaria Integrata S. Maria della Misericordia, 33100 Udine, Italy

**Keywords:** metastatic colorectal cancer, biomarkers, anti-EGFR agents, primary resistance, negative selection

## Abstract

In the evolving molecular landscape of metastatic colorectal cancer, optimizing available tools to select patients to receive anti-epidermal growth factor receptor (anti-EGFR) monoclonal antibodies is a modern challenge of colorectal oncologists. Several molecular biomarkers have been investigated in recent years as potential predictors of resistance to anti-EGFR agents in preclinical and clinical retrospective series. Nevertheless, none of them have been implemented in clinical practice due to the lack of a formal prospective demonstration. Here, we propose a literature review of molecular alterations associated with resistance to anti-EGFRs, underlining the reasons why their roadmap from laboratories to clinics was prematurely halted.

## 1. Introduction

Epidermal Growth Factor Receptor (EGFR) is the first discovered member of the family of ErbB tyrosine kinase receptors. Its activation results in a wide variety of transduction events through the downstream RAS-RAF-MEK-ERK signaling pathway, able to control cell proliferation, migration, and survival. Aberrant EGFR activation in tumor cells can result from increased transcriptional expression and/or gene amplification or activating mutation. Increased EGFR protein and transcript levels correlate with poor prognosis in various epithelial cancers, including colorectal cancer (CRC).

Targeting Epidermal Growth Factor Receptor by means of monoclonal antibodies (moAbs) (i.e., cetuximab and panitumumab) that inhibit endogenous ligands’ binding and thus lead to the inhibition of downstream signaling pathways, both as monotherapies or in combination with a chemotherapy backbone, allowed achieving clinically relevant therapeutic advances in metastatic colorectal cancer (mCRC) patients [1,2,3,4].

In the last decade, the optimization of the use of anti-EGFR agents in mCRC has acquired growing remark within the field. From this perspective, a substantial improvement of the cost/benefit ratio was provided by the identification of *RAS* activating mutations, involving Kirsten Rat Sarcoma viral oncogene homolog (*KRAS*) and Neuroblastoma RAS viral oncogene homolog (*NRAS*) codons 12 and 13 of exon 2, 59 and 61 of exon 3, and 117 and 146 of exon 4, as predictors of intrinsic resistance to EGFR blockade [5,6].

As a consequence, *RAS* mutational status is today the only molecular marker taken into account by all current international guidelines and regulatory agencies to negatively select mCRC patients to an anti-EGFR-based treatment [7,8], thus leading to exclusion of those with *RAS*-mutated tumors from receiving EGFR inhibitors. 

Nevertheless, even selecting patients with *RAS* wild-type disease, only a limited percentage of them derives benefit from anti-EGFR agents, thus suggesting the emerging need to disclose molecular mechanisms, other than *RAS*, underpinning the primary resistance to EGFR blockade and introducing one of the currently most intriguing and hard challenges of precision medicine.

Retrospective findings from clinical studies, supported by a biological rationale, have suggested a potential clinical interest for some molecular alterations which seem to negatively affect tumor susceptibility to EGFR inhibition. However, to date, none of these biomarkers have produced a sufficient level of evidence to enter clinical practice.

**Figure 1:**
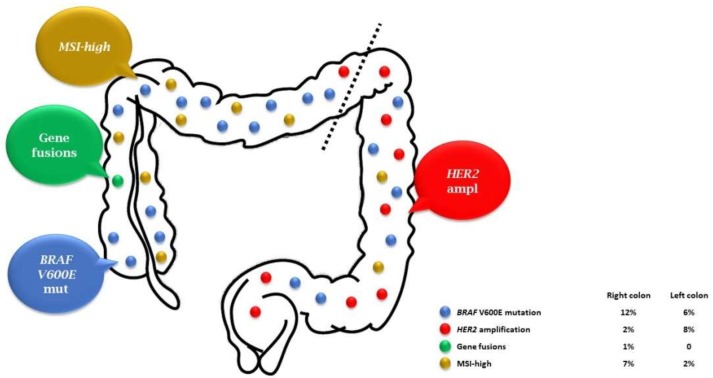
Distribution of molecular alterations in *RAS* wild-type mCRC, according to primary tumor location (the separation between right- and left-sided colon is indicated by the dotted line).

Herein, we propose an updated literature review of the available evidence on molecular biomarkers (Figure 1), other than *RAS* status, which recently started their long winding roadmap toward implementation in clinical practice.

## 2. *BRAF* V600E Mutation: Going beyond Formal Statistical Demonstrations

V-raf murine sarcoma viral oncolgene homolog b1 (*BRAF*) mutations occurring in codon 600 affect about 8–10% of mCRC patients [9,10] and are mutually exclusive with *RAS* mutations (Figure 1). *BRAF* V600E-mutated mCRC share distinctive clinical and pathological features: they are more common in women and elderly patients, are often right-sided, present mucinous histology and microsatellite instability, and have a frequent dissemination to lymph nodes and peritoneum [11]. Furthermore, *BRAF* V600E mutation is associated with extremely poor prognosis across all stages of disease [12,13] and has been recently depicted as a key genomic marker of two consensus transcriptomic subtypes (CMSs) of colon cancer, CMS1 and CMS3 [14]. 

Due to this well-known negative impact on survival, all current guidelines strongly recommend to adopt *BRAF* status as a stratification factor for clinical trials in the metastatic setting [7,8]. Moreover, *BRAF* V600 analysis is recommended for the risk assessment for Lynch Syndrome in CRC patients with microsatellite instable (MSI-high) tumors [8,15].

The potential role of *BRAF* V600E mutation as a negative predictor of benefit from anti-EGFR moAbs has been widely investigated both in preclinical studies [6,16], which corroborated the biological rationale, and in large clinical series reporting no response to anti-EGFR as monotherapy in the chemo-refractory setting [17,18,19,20].

In addition, data from two meta-analyses, including results from key clinical trials testing the addition of an anti-EGFR to standard chemotherapy regimens or best supportive care (BSC) in *BRAF* wild-type and *BRAF* V600E-mutated mCRC, showed that the addition of an anti-EGFR in *RAS* and *BRAF* wild-type tumors provides a clear benefit, whereas the impact in *BRAF* V600E-mutant disease is limited or null [21,22], although the interaction effect between anti-EGFR treatment and *BRAF* mutational status was not statistically significant, especially in terms of overall survival [22].

Drawing from these results, even in the absence of a formal demonstration, considering the minimal, although not detrimental, impact of anti-EGFRs in *BRAF* V600E-mutated disease, these patients are generally not treated with cetuximab or panitumumab at least in the first-line setting. 

A clinically significant improvement in the outcome of this molecularly defined subset of mCRC has been reported in three clinical experiences adopting a more intensive first-line treatment, the triplet FOLFOXIRI (including 5-fluoruracil, oxaliplatin and irinotecan) plus bevacizumab [23,24,25]. Results achieved in terms of activity and efficacy led to the hypothesis that FOLFOXIRI plus bevacizumab may be able to counteract the intrinsic biological aggressiveness of this poor prognosis disease and, as a consequence, is today recognized by international guidelines as a preferred treatment option for selected patients with *BRAF* V600E-mutant mCRC [7,8]. 

The acknowledgement of the role of *BRAF* V600E mutation as an oncogenic driver pushed the development of targeted approaches [26]. After initial disappointing results of combining *BRAF* and *MEK* inhibitors, more encouraging preliminary data have been reported by clinical trials evaluating triple-drug combinations of anti-EGFR moAbs, *BRAF* inhibitors, and a PI3K*α* inhibitor or a MEK inhibitor, with more convincing results than “targeted doublets” (i.e., EGFR and BRAF inhibitors) [27,28,29,30].

## 3. Atypical *RAS* and *BRAF* Mutations: What Do They Mean?

Advances in technologies for gene sequencing currently allow comprehensively testing of multiple mutational hotspots within large panels of genes of clinical interest [31,32]. These wide genomic analyses are able to provide a huge amount of information about rare molecular alterations, most of them with completely unknown biological and clinical meaning. That has been the case of “atypical” *RAS* and *BRAF* mutations, mapping outside of the codons conventionally tested and with well-known predictive impact. 

With regard to *RAS* mutations occurring outside those codons that must be tested according to the current labels of both cetuximab and panitumumab, limited retrospective data showed that a subset of them has lower median downstream signaling activity, compared to typical ones [33]. The clinical meaning of these alterations, with special regard to their predictive impact, is not yet elucidated.

Two retrospective series described *BRAF* non-V600E mutations in a small percentage of mCRCs (prevalence around 2%) [34,35]. Mainly occurring at codons 594 and 596, these mutations define a specific molecular subtype of mCRC with good prognosis, similar to that of *BRAF* wild-type cases, and clinical and molecular features opposite to those of *BRAF* V600E-mutated tumors. Indeed, they are more common in young males, left-sided and microsatellite stable tumors, with possibly coexisting *RAS* mutations [34,35,36]. The peculiar phenotype and clinical behavior is consistent with preclinical evidence describing a kinase inactivating effect of these mutations [37].

The functional characterization of mutations involving not routinely tested codons of *RAS* and *BRAF* genes, together with their potential interference with the sensitivity to anti-EGFR agents and consequent therapeutic implications in the management of mCRC patients, warrants further investigations. 

## 4. *HER2*: Preliminary Retrospective Evidence

In the last few years, clinical interest has surrounded human epidermal growth factor receptor 2 (*HER2*) amplification not only as an oncogenic driver but also as a new target in mCRC and a potential predictor of resistance to EGFR inhibitors (Table 1).

In fact, preclinical observations firstly highlighted the association of this molecular alteration with lack of sensitivity to anti-EGFR moAbs [39]. By taking advantage of a large library of xenografts derived from mCRC patients (i.e., xenopatients), *HER2* amplification was detected in *RAS* and *BRAF* wild-type anti-EGFR-resistant xenopatients and thus supposed as a mechanism of intrinsic resistance to EGFR inhibition [38].

In the attempt to identify potential novel treatment options for this molecularly defined subgroup of CRCs, mice bearing *HER2*-amplified, anti-EGFRs resistant patient-derived mCRC xenografts were treated with various single or combined *HER*2-targeted drugs, showing a more pronounced sensitivity to *HER*2-blockade with trastuzumab in combination with lapatinib, but not to either agent alone [38].

These promising preclinical observations paved the way for the design of clinical trials testing anti-*HER*2 strategies in refractory mCRC patients. The phase II HERACLES-A trial was a single-arm proof-of-concept study assessing the activity of the dual *HER*2-targeted inhibition with trastuzumab and lapatinib in a cohort of 33 patients with *HER2*-amplified, *KRAS* wild-type mCRC who failed standard-of-care treatments, including cetuximab or panitumumab. The HERACLES-A trial met the primary endpoint, achieving an overall response rate of 30% in a setting of heavily pre-treated patients [45]. Similarly, the phase II MyPathway multiple basket trial confirmed the exceptional activity of combining two anti-*HER*2 agents, trastuzumab and pertuzumab, obtaining an overall response rate of 38% in the same setting of disease (i.e., patients with *HER2*-amplified/overexpressed mCRC who had exhausted standard treatments) [46]. 

Together with the above-mentioned preclinical evidence, a number of retrospective clinical series clearly supported that activation of *HER*2 signaling determines resistance to cetuximab or panitumumab (Table 1). Survival outcomes of patients treated with anti-EGFR were negatively influenced by *HER2* amplification: median PFS on anti-EGFR therapy was significantly shorter for patients harboring *HER2*-amplified compared to non-amplified tumors [41,42]. Moreover, in the HERACLES-A study, conducted exclusively in *HER2*-positive mCRC patients, those who had been previously treated with panitumumab or cetuximab were resistant to such therapies [45].

In spite of the reproducibility of these findings across different experiences worldwide, the translation of *HER2* amplification/overexpression as a predictive marker in the daily practice is hampered by the lack of anti-EGFR-untreated control groups in previously mentioned series, as well as by the lack of prospective results. On the other hand, the reliability of *HER2* evaluation by means of an easy-to-perform immunohistochemistry and the definition of well-established criteria for its interpretation would make the way towards clinical application quite simple for this marker.

## 5. How to Deal with Very Rare Alterations? The Example of Gene Fusions

Translocation and rearrangements involving anaplastic lymphoma kinase (*ALK*), ROS proto-oncogene 1 (*ROS1*), neurotrophic tyrosine kinase receptor 1-2-3 (*NTRK1-3*), and rearranged during transfection (*RET*) genes are rare molecular events inducing a constitutive activation of tyrosine kinase receptors, leading to enhanced cellular proliferation, differentiation, and survival in a wide range of solid malignancies [47,48]. These molecular alterations with a potential driver impact were recently described in a small fraction of mCRCs [49,50,51], with an overall incidence in the range of 0.5–2% [52,53] (Figure 1).

In the context of an international collaborative effort, the clinical and molecular landscape of *ALK*, *ROS1*, *NTRK*, and *RET* rearranged mCRC has been recently deepened. Sharing some features with *BRAF* V600E mutation, *ALK*, *ROS1*, *NTRK*, and *RET* rearrangements more frequently occur in elderly patients, right-sided tumors, *RAS* wild-type, and MSI-high cancers [54,55]. Furthermore, gene fusions confer a strong negative impact on survival, independent of other prognostic characteristics.

Encouraging results suggest that patients with mCRC bearing these genomic alternations may benefit from therapeutic targeted approaches with small molecules that selectively inhibits tropomyosin receptor kinase (Trk) A-B-C, ALK, ROS1 (encoded by the *NTRK1-2-3*, *ALK*, and *ROS1* genes), such as entrectinib, or only TrkA-B-C or RET, such as larotrectinib and LOXO-292 [56,57,58,59,60,61,62], respectively. On the other hand, a robust biological rationale supported by preclinical in vitro findings seems to suggest a low EGFR-dependency of these rearranged tumors [47], confirmed also in small retrospective studies, in which the limited subset of patients with rearrangement-positive disease derived no benefit from anti-EGFR moAbs [54,55]. This observation should encourage clinicians to avoid EGFR inhibition and to adopt targeted approaches as soon as possible in the disease course of these patients. While the level of produced evidence with regard to the negative predictive impact of gene fusions is very low, their rarity makes their reproduction on a large scale extremely hard.

## 6. Looking for Prospective Evidence: The Case-Control PRESSING Study

In order to overcome the intrinsic limitations of retrospective studies, and to move above commented markers towards the clinical practice, a prospective case-control study, named PRESSING (PRimary rESiStance IN *RAS* and *BRAF* wild-type metastatic colorectal cancer patients treated with anti-EGFR monoclonal antibodies), was recently conducted. The objective of the study was to prospectively validate the negative predictive impact of a panel of rare genomic alterations, on the basis of a pre-planned translational hypothesis. *RAS* and *BRAF* wild type patients were included among cases (resistant patients) and controls (sensitive patients), respectively, if they had experienced rapid disease progression or clear benefit from anti-EGFR treatment. In order to avoid the confounding effect of the associated chemotherapy backbone, only patients treated with anti-EGFR monotherapy or with an anti-EGFR plus irinotecan (only if irinotecan-refractory) were included. The following genomic alterations were comprised in the so-called PRESSING panel: *HER2* and MET proto-oncogene (*MET*) amplifications, *ALK*, *ROS1*, *NTRK1-3*, and *RET* fusions, and *HER2*, phosphoinositide 3-Kinase (*PI3K*), phosphatase and TENsin homolog (*PTEN*), protein kinase B (*AKT1*) mutations. The trial met its primary endpoint, showing a significantly higher prevalence of negative predictors of benefit from anti-EGFRs among resistant than among sensitive patients, thus opening the way to a new concept of “negative hyperselection” of patients to be treated with this class of drugs. Notably, the PRESSING panel was able to unveil mechanisms of primary resistance in around half of rapidly progressing patients [44].

Recent post-hoc analyses of randomized trials underline that primary location affects the sensitivity to anti-EGFRs [63]. In particular, in terms of predictive effect, while a clear benefit from these drugs was observed in left-sided tumors, no significant benefit for right-sided ones was reported. This was supported by biological background, considering a higher prevalence of molecular mechanisms potentially driving intrinsic resistance to anti-EGFRs in right-sided tumors, and is confirmed also in the PRESSING series. Indeed, the PRESSING panel alterations were significantly more common in proximal tumors. From a clinical perspective, the combined evaluation of primary tumor location and this panel of candidate genomic alterations in *RAS* and *BRAF* wild-type patients may allow excluding a substantial proportion of resistant patients from EGFR inhibitors with meaningful predictive accuracy, thus representing a step forward in the way towards the optimization of the use of this therapeutic targeted approach. 

## 7. New Biomarkers on the Horizon? Focus on Microsatellite Instability and Consensus Molecular Subtypes

Whereas microsatellite instability has recently emerged as a positive biomarker for the selection of mCRC patients who benefit from immunotherapeutic agents, such as pembrolizumab and nivolumab [64,65,66], preliminary data put it on the horizon of molecular markers with a supposed impact on tumor sensitivity to EGFR inhibition. In the post-hoc analysis of phase III randomized CALGB/SWOG 80405 trial, patients with MSI-high tumor had a clear worse outcome when receiving a cetuximab-based than a bevacizumab-based first-line therapy [67]. Moreover, in the above-mentioned PRESSING study, microsatellite instability was significantly more frequent in anti-EGFR-resistant than sensitive patients, and associated with other predictors of primary resistance and with right-sidedness [44].

The high tumor mutational burden, typical phenotype of MSI-high tumors, could activate multiple oncogenic signals and thus negatively interfere with the therapeutic inhibition of a single pathway, i.e., EGFR blockade. However, considering their retrospective nature, these suggestions about MSI-high as a determinant of resistance to anti-EGFRs should be uniquely regarded as hypothesis-generating and thus should be further investigated. 

In addition, a recent international effort resulted in the categorization of the heterogeneity of CRC at gene-expression level into four biologically homogeneous consensus molecular subtypes (CMSs) [14]. Each CMS is characterized by distinct gene expression profiles and well-defined genomic and epigenomic key features, intimately linked to the cellular phenotype and tumor clinical behavior [68]. Although the CMS classification is manly trained on complex transcriptomic patterns and developed as a stratification tool with a clear prognostic impact across all stages of CRC disease [14], these transcriptomic subtypes have been tested also for a potential predictive value with regard to targeted agents. Indeed, based on data from preclinical models, the CMS2 subtype (i.e., “canonical subtype”, characterized by epithelial differentiation and marked expression of a number of oncogenes, among them EGFR, HER2, insulin-like growth factor 2 (IGF2), insulin receptor substrate 2 (IRS2) and transcription factor hepatocyte nuclear factor 4α (HNF4A)) seemed to have a stronger sensitivity to anti-EGFR agents compared to other CMSs [69]. However, the preclinically suggested ability of CMS subtypes to predict the benefit from targeted agents has not been confirmed in post-hoc analyses of randomized phase III trials comparing bevacizumab- versus cetuximab-based first-line therapy for mCRC patients [70,71].

Taking in account that these data on CMSs derive from retrospective analyses and are not perfectly consistent among different series, further studies are warranted to understand whether and how this biological gene expression-based classification of CRC could have a potential utility in the clinical scenario.

## 8. Personalizing the Use of Anti-EGFRs: A Potential Application of Liquid Biopsy

Liquid biopsy provides a non-invasive approach to analyse the tumor genomic landscape from blood-derived circulating cell-free tumor DNA (ctDNA). Its potential application to optimize and personalize the management of CRC patients at different stages of disease is attracting growing interest. As compared to the test of a tissue biopsy, able to catch tumor characteristics in a defined space and timeframe, the liquid biopsy shows several advantages, including being a noninvasive procedure, having a fast turnaround time, and the ability to provide a more comprehensive portrait of spatial and temporal intratumor heterogeneity.

Several proof-of-concept and mainly retrospective experiences highlighted the potential role of liquid biopsy as a tool to optimize the use of anti-EGFRs in clinical practice. Indeed, liquid biopsies might be used to test *RAS* status in ctDNA instead of in tissue biopsy, to monitor the efficacy of anti-EGFR agents by tracking early mechanisms of acquired resistance during anti-EGFR-containing treatments, to evaluate the potential usefulness of rechallenge with anti-EGFRs.

The accuracy of *RAS* testing in ctDNA has not been fully elucidated in mCRC patients, due to the lack of standardized ctDNA assays. Head-to-head retrospective series reported a concordance rate higher than 90% between results of tissue and plasma analyses [72,73,74,75,76,77,78]. Noteworthy, not only intrinsic analytical factors, but also a number of clinical and pathological variables, including sites of metastases, disease burden, and tumor histology, may influence the release of ctDNA by tumor cells, thus affecting results of plasma testing.

With regard to the potential application of liquid biopsy in longitudinally monitoring treatment efficacy and resistance, the emergence of *RAS* mutations is a well-recognized mechanism of secondary resistance to anti-EGFR moAbs. The rise of *RAS* mutated alleles in ctDNA at the time or even before the evidence of disease progression was described more than five years ago. From that moment on, several research groups produced very heterogeneous results with regard to the percentage of cases in which disease progression is actually driven by these and other molecular events (*BRAF*, *EGFR*, and *PIK3CA* mutations, *HER2* and *MET* amplification) [39,79,80,81,82,83].

Finally, translational analyses from a recent phase II single arm study [84] showed that among patients with *RAS* and *BRAF* wild-type tumors with acquired resistance to first-line chemotherapy plus cetuximab, only those with *RAS* and *BRAF* wild-type ctDNA at the time of cetuximab rechallenge could potentially derive benefit from this strategy. Based on these results, prospective trials are warranted in order to validate this hypothesis and to prompt the translation of liquid biopsy from the lab to clinical practice.

## 9. Levels of Evidence and Pragmatic Approaches: How to Make the Roadmap Less Winding?

In current daily practice, the molecular selection of patients to receive anti-EGFR monoclonal antibodies is based on the exclusion of those with tumors bearing *RAS* and *BRAF* mutations. Nevertheless, since only around 50% of patients with *RAS* and *BRAF* wild-type tumors do achieve a response when treated with anti-EGFRs, several rare genomic alterations leading to the activation of tyrosine kinase receptors other than EGFR or downstream signaling pathways have been proposed as predictors of intrinsic resistance to anti-EGFR agents. However, despite coherent preclinical and retrospective suggestions, most of them have early interrupted their winding roadmap from bench to bedside, since the reliability of each candidate marker has not been prospectively challenged in the most updated clinical and molecular scenario. At the same time, due to the low prevalence of these molecular alterations, conducting proper prospective validation studies or post-hoc analyses of randomized clinical studies aiming to assess the impact of each marker would be unrealistic. Drawing from these considerations and proposing a pragmatic approach to tackle these limitations, an academic prospective case-control study has recently demonstrated the clinical utility of a panel of uncommon genomic alterations, including *HER2* and *MET* amplifications, *ALK*, *ROS1*, *NTRK1-3*, and *RET* fusions, and *HER2*, *PI3K*, *PTEN*, *AKT1* mutations, in predicting intrinsic resistance to anti-EGFR agents in molecularly (i.e., *RAS* and *BRAF* wild-type) selected patients [44], thus introducing the concept of “negative hyperselection”. Moreover, these findings were embedded in the debated scenario of right versus left primary location. By one side, the usefulness of primary sidedness as a surrogate marker of the much more complex landscape of molecular features underpinning the different behavior or right- and left-sided tumors was corroborated. By the other side, the combined evaluation of primary sidedness and genomic alterations was able to provide the best predictive accuracy. 

Of note, among negatively hyper-selected mCRC patients (i.e., patients with *RAS* and *BRAF* wild-type disease, and not bearing any of the alterations included in the PRESSING panel), a proportion of them still derive no benefit from anti-EGFR agents, by underlying that the negative hyper-selection would be further refined by investigating non-genomic mechanisms potentially affecting the sensitivity to the EGFR inhibition.

From this perspective, as a next step to unveil the complex molecular landscape underpinning primary resistance to EGFR blockade beyond *RAS* and *BRAF* mutations and “PRESSING panel” genomic alterations, a prospective case-control study has been recently launched to assess the negative predictive role of a panel of gene expression profiles in two independent cohorts of patients with *RAS* and *BRAF* wild-type and “PRESSING panel” negative mCRC treated with anti-EGFR agents. The cohort of cases will include resistant patients and controls of those who clearly benefit from anti-EGFR moAbs.

In addition, as the predictive accuracy of the combined evaluation of primary tumor sidedness and “PRESSING panel” assessment in predicting the anti-EGFR treatment outcome is around 80%, the “positive” identification of patients with mCRC characterized by a real EGFR pathway-addiction is now extremely appealing. As a consequence, genomic markers (such as Insulin Receptor Substrate 2 (*IRS2*) amplifications or mutations, EGFR amplification, amphiregulin (*AREG*) and epiregulin (EREG) amplification), and non-genomic mechanisms preclinically related to sensitivity to anti-EGFRs will be evaluated among patients who derived clear benefit from an anti-EGFR-containing strategy. The occurrence of some common secondary effects of anti-EGFR agents, including skin rash, hypomagnesemia, or xerosis, is postulated to serve as an early response predictor in mCRC patients [85,86]. These initial observations have never been prospectively validated and underlying mechanisms have never been elucidated.

Is this level of evidence enough to translate “PRESSING panel” biomarkers from theory into the daily practice? Most clinicians and methodologists will negatively answer this question, considering retrospective evidence and a prospective but not randomized trial as insufficient proof to change clinical attitudes.

At the same time, the rarity of these alterations definitely undermines the feasibility of large randomized trials, so that the level of provided evidence will hardly increase towards commonly accepted standards.

In this controversial situation, *HER2* amplification/overexpression is among the abovementioned markers closest to entering the molecular workup of mCRC patients, based on coherent preclinical evidence and consistent data from retrospective and prospective clinical studies. Moreover, unlike all other markers of intrinsic resistance to anti-EGFRs that are more prevalent in right-sided tumors, *HER2* amplification/overexpression is more represented in *RAS* and *BRAF* wild-type distal tumors, where its frequency is as high as 8–10% (Figure 1). Since patients with left-sided *RAS* and *BRAF* wild-type tumors are currently the optimal candidates to receive a first-line anti-EGFR-containing therapy, *HER2* amplification/overexpression provides clear added value to the selection based on *RAS* and *BRAF* status and primary tumor location, by identifying a molecularly defined subset of patients in which the use of anti-EGFR agents should be avoided. Furthermore, the recognition of this marker would allow not only preventing a futile and potentially toxic approach with anti-EGFRs, but also offering early access to novel treatment options in the frame of properly designed clinical trials. While the availability of dedicated approaches for MSI-high tumors clearly highlights the need to treat these patients with immunotherapic agents, the frequent co-occurrence of other genomic alterations makes the added value of this marker as a negative predictor of benefit from anti-EGFRs less clear.

Novel insights in the molecular scenario of mCRC could derive from the molecular pathological epidemiology (MPE), a developing multidisciplinary field investigating whether exposure factors (i.e., lifestyle, environmental, or genetic factors) are associated with specific molecular alterations (i.e., *RAS* and *BRAF* mutations, microsatellite status, etc.). MPE also addresses whether a specific genomic feature could interact with a particular exposure factor to affect tumor prognosis and response to specific treatments [87]. Although nowadays no validated relationship between a certain exposure and a specific molecular marker is recognized, MPE could contribute to personalized prevention strategies and treatment choices [88]. Obviously, due to the retrospective nature of this field of research, several biases could affect the findings and limit their inference [87].

In conclusion, advances in the molecular characterization of mCRC have opened the way to novel markers potentially affecting the efficacy of anti-EGFR agents. Although, in the absence of a formal demonstration of its negative predictive impact, *HER2* amplification/overexpression might be taken into account to improve the selection of patients to this class of drugs.

## Figures and Tables

**Table 1:** Studies investigating *HER*2 amplification as a predictor of resistance to anti-EGFR agents.

Reference	Study Design	Population	Main Results
Bertotti et al. [38]	Preclinical	85 xenopatients ^a^, expanded in two molecularly unselected cohorts; randomized to receive or not cetuximab	*HER2* amplification or overexpression in 6 cases out of 44 *KRAS* wild-type patients resistant to anti-EGFR vs. 0 out of 45 *KRAS* wild-type patients with objective response to anti-EGFR (*p* < 0.05)
Yonesaka et al. [39]	Retrospective	182 *KRAS* wild-type patients treated with cetuximab-based therapy	Worse outcome (PFS and OS) for patients with *HER2*-amplified vs. *HER2*-nonamplified tumors
Martin et al. [40]	Retrospective	162 *KRAS* wild-type patients treated with anti-EGFR	Worse outcome (RR, PFS and OS) for patients with *HER2* FISH^+^ vs. *HER2* FISH^−^ tumors
Raghav et al. [41]	Retrospective	196 *RAS* and *BRAF* wild-type mCRC patients treated with anti-EGFR therapy	Worse outcome (PFS) for patients with *HER2*-amplified vs. *HER2*-nonamplified tumors
Sartore-Bianchi et al. [42]	Retrospective	80 patients with *HER2*-amplified and *KRAS* wild-type tumors	Worse outcome (RR and PFS) for patients treated with anti-EGFR vs. patient not treated with anti-EGFR
Sawada et al. [43]	Retrospective	11 patients with *HER2*-amplified and *RAS* and *BRAF* wild-type tumors	Worse outcome (RR, PFS and OS) for patients with *HER2*-amplified and *RAS/BRAF* wild-type vs. *HER2*-nonamplified and *RAS/BRAF* wild-type tumors
Cremolini et al. [44]	Prospectivecase-control	94 *RAS/BRAF* wild-type patients: 47 patients resistant and 47 patients sensitive to anti-EGFR-based therapy	*HER* amplification in 7 cases out of 47 resistant patients vs. 0 out 47 sensitive patients (*p* = 0.01)

^a^ human cancer specimens directly transplanted into mice. FISH: Fluorescent in situ hybridization. RR: Response Rate; PFS: Progression-free survival; OS: Overall survival.

## Abbreviations

AKT1	Protein Kinase B
ALK	Anaplastic Lymphoma Kinase
AREG	Amphiregulin
BRAF	V-raf Murine Sarcoma Viral Oncogene Homolog b1
BSC	Best Supportive Care
CMS	Consensus Molecular Subtypes
EGFR	Epidermal Growth Factor
EREG	Epiregulin
HER2	Human Epidermal Growth Factor Receptor 2
HNF4A	Hepatocyte Nuclear Factor 4α
IGF2	Insulin-Like Growth Factor 2
IRS2	Insulin Receptor Substrate 2
KRAS	Kirsten Rat Sarcoma Viral Oncogene Homolog
mCRC	Metastatic Colorectal Cancer
moAbs	Monoclonal Antibodies
MET	MET Proto-Oncogene
MPE	Molecular Pathological Epidemiology
NRAS	Neuroblastoma RAS Viral Oncogene Homolog
NTRK 1–3	Neurotrophic Tyrosine Kinase Receptor 1–3
OS	Overall Survival
PI3K	Phosphoinositide 3-Kinase
PFS	Progression-Free Survival
PTEN	Phosphatase and TENsin Homolog
RET	Rearranged during Transfection
ROS1	ROS Proto-Oncogene 1
RR	Response Rate
Trk	Tropomyosin Receptor Kinase
